# Automatic classification of hyperkinetic, tonic, and tonic-clonic seizures using unsupervised clustering of video signals

**DOI:** 10.3389/fneur.2023.1270482

**Published:** 2023-11-02

**Authors:** Petri Ojanen, Csaba Kertész, Elizabeth Morales, Pragya Rai, Kaapo Annala, Andrew Knight, Jukka Peltola

**Affiliations:** ^1^Faculty of Medicine and Health Technology, Tampere University, Tampere, Finland; ^2^Neuro Event Labs, Tampere, Finland; ^3^Department of Neurology, Tampere University Hospital, Tampere, Finland

**Keywords:** epilepsy, seizure classification, motor seizures, signal analysis, biomarkers

## Abstract

**Introduction:**

This study evaluated the accuracy of motion signals extracted from video monitoring data to differentiate epileptic motor seizures in patients with drug-resistant epilepsy. 3D near-infrared video was recorded by the Nelli^®^ seizure monitoring system (Tampere, Finland).

**Methods:**

10 patients with 130 seizures were included in the training dataset, and 17 different patients with 98 seizures formed the testing dataset. Only seizures with unequivocal hyperkinetic, tonic, and tonic-clonic semiology were included. Motion features from the catch22 feature collection extracted from video were explored to transform the patients' videos into numerical time series for clustering and visualization.

**Results:**

Changes in feature generation provided incremental discrimination power to differentiate between hyperkinetic, tonic, and tonic-clonic seizures. Temporal motion features showed the best results in the unsupervised clustering analysis. Using these features, the system differentiated hyperkinetic, tonic and tonic-clonic seizures with 91, 88, and 45% accuracy after 100 cross-validation runs, respectively. F1-scores were 93, 90, and 37%, respectively. Overall accuracy and f1-score were 74%.

**Conclusion:**

The selected features of motion distinguished semiological differences within epileptic seizure types, enabling seizure classification to distinct motor seizure types. Further studies are needed with a larger dataset and additional seizure types. These results indicate the potential of video-based hybrid seizure monitoring systems to facilitate seizure classification improving the algorithmic processing and thus streamlining the clinical workflow for human annotators in hybrid (algorithmic-human) seizure monitoring systems.

## 1. Introduction

Overall, 30% of patients diagnosed with epilepsy suffer from uncontrolled seizures despite the adequate use of anti-seizure medications (ASM) (1). Drug-resistant epilepsy (DRE) causes an increased risk of mortality and morbidity (2) and sudden unexplained death in epilepsy (SUDEP) (3). Accurate seizure documentation is essential to optimize the treatment of epilepsy. Previous research studies have demonstrated inaccuracies related to seizure diaries (4, 5), which has given an impetus for the development of various seizure detection systems, aiming for more objective seizure documentation. Though seizure detection systems have improved seizure documentation, seizure classification based on videos or other data can still be challenging (6–8).

The International League Against Epilepsy (ILAE) has recently published new guidelines for the classification of epileptic seizures (9). ILAE seizure classification categorizes seizures based on their focal or generalized onset, level of awareness, and non-motor and motor manifestations. Seizures can also be classified based on semiology, only highlighting the relevance of the observable ictal motor and other manifestations without electrophysiological information from EEG. In semiological classification, motor manifestations are depicted as simple or complex based on the complexity of the movement (10–12). Laterality (left, right, or bilateral) and chronological order of the symptoms are additional classification features (10, 13).

Video-based methods in the detection of epileptic seizures have been widely studied with high sensitivity and specificity for detection performance (14). Studies have shown promising results in the analysis of semiological features by utilizing convolutional neural networks (CNN) and long short-term memory (LSTM) in facial and body movement analysis (15), deep learning methods (16), and movement trajectories (17) in body movement analysis and ictal sound recordings in seizure semiology analysis (18). However, automatic seizure classification is a less explored topic. Temporal lobe epilepsy (TLE) and frontal lobe epilepsy (FLE) have been differentiated by utilizing movement trajectories (19) or quantitative movement analysis (20). Infrared and depth sensors were used in 3D video data to differentiate between seizures in FLE, TLE, and non-epileptic events reaching a cross-subject f1-score (a metric to assess machine-learning predictive skill) of 0.833 when differentiating between FLE and TLE seizures and of 0.763 when differentiating between FLE, TLE, and non-epileptic events from each other (21). However, only a few studies have evaluated the performance of deep learning in the analysis of multiple distinct motor seizure types.

The Nelli seizure monitoring system is an audio/video-based semi-automated (hybrid) seizure monitoring platform that uses computer vision and machine learning to identify kinematic data (motion, oscillation, and audio) commonly associated with seizures with a positive motor component and human experts to visually assess these epochs (22). Moreover, the utility of the hybrid (algorithm-human) system for reviewing nocturnal video recordings to significantly decrease the workload and to provide accurate classification of major motor seizures (tonic-clonic, clonic, and focal motor seizures) has been demonstrated (23). The potential to differentiate seizure types by utilizing algorithmic signal profiles was first explored in a previous case study (24). Even though Nelli^®^'s algorithmic performance in seizure detection has been demonstrated in previous validation studies (25), the potential of the algorithmic part of the system to classify seizure types has not been previously explored.

Given the potential of deep-learning methods to differentiate seizure types and the need for a tool to assist in seizure classification, novel methods to classify specific seizure types using video monitoring and deep learning are needed. One recent development on this frontier has been the catch22 feature collection (26). The catch22 project has implemented over 7,700 time-series features from multiple science fields to find the best-performing statistics for time-series classification, finally selecting the top 22 features for their software library to perform feature extraction or dimension reduction for time-series analysis. These features have been applied successfully in a wide range of scientific problems: e.g., tree deformation detection in winds (27), hydroclimatic data processing (28), human breast cancer cell detection (29), commercial sales prediction (30), or cardiometabolic risk detection (31). They have not been previously applied for video-based seizure classification.

The aim of this study was to evaluate the performance of a novel signal algorithm model in classifying tonic, tonic-clonic, and hyperkinetic seizures by utilizing motion and oscillation signal profiles. This study further examines the previously recognized potential of the Nelli system to automatically classify aforementioned seizure types by utilizing signal profiles and deep learning to take a step toward automatic seizure classification.

## 2. Methods

### 2.1. Patient population

A total of 27 patients with focal DRE were enrolled in the study. The study protocol and informed consent forms were reviewed and approved by the ethics committee of Tampere University Hospital. Signed informed consent was obtained from each participant. All patients were on two or more ASMs, and some of them were also treated with vagal nerve stimulation (VNS) therapy. Each patient was monitored from 4 to 8 weeks in a home setting for 7–11.5 h per night (average 9.19 h, median 9.25 h). Unequivocal seizures from previous recording sessions of enrolled patients were utilized only if they lacked unequivocal seizures in the latest monitoring period. Training patients were selected partly from a recent interventional study (22) and partly from Nelli^®^ post-market surveillance (PMS) recordings, and testing patients were selected from Nelli^®^ PMS recordings with the requirement that, for each subject, at least three unequivocal seizures of these three seizure types of interest were recorded during Nelli^®^ registration and they had been described in detail in previous video-EEG reports. Due to the exclusion criteria listed above, 130 seizures from 10 patients formed a cohort for the model training, including four patients from the previous study (22). The testing patient cohort consisted of 98 seizures from 17 patients, who were not included in the training phase, to evaluate the performance of the model. Patient demographics and seizure counts are presented in Table 1.

**Table 1:** Patient demographics and clinical characteristics.

**Characteristics**	**Training phase (*n =* 10)**		**Testing phase (*n =* 17)**	
Age range (years)	18–46		18–58	
Mean age (years)	34.5		33.8	
**Gender**				
Male	5 (50%)		10 (58.8%)	
Female	5 (50%)		7 (41.2%)	
**Seizure type**	**Training phase**		**Testing phase**	
	**Patients**	**Seizures**	**Patients**	**Seizures**
Tonic-clonic	4	12	3	6
Hyperkinetic	5	73	7	41
Tonic	3	44	7	51
**Total**	**10** ^*^	**129**	**17**	**98**

^*^Two patients contributed more than one seizure types.

### 2.2. Video monitoring

Video monitoring was performed by NEL (Neuro Event Labs, Tampere, Finland) using the Nelli^®^ seizure monitoring system consisting of a camera and a microphone installed at the patient's bedside in their home so that the patient stays in sight of the camera during periods of rest. Video data from all patients were manually annotated. The epochs of suspected seizure events were reviewed by expert epilepsy annotators. Previous VEM (video-EEG monitoring) reports obtained before the start of the study were used for the assessment of behavioral features of seizures that occurred during Nelli monitoring. Seizures were classified by professionals according to the ILAE 2017 classification (9). Suspected seizure events were excluded from further analysis if they were not unequivocally identified as seizures by comparing them to previous VEM reports which were considered a feasible reference standard for the phase two study as previously suggested (32). Seizures were considered unequivocal to a seizure type if they were identified based on VEM reports and they shared similar manifestations as described in classification guidelines. All seizures belonging to the hyperkinetic, tonic, or tonic-clonic seizure type categories were included. These three seizure types were the most common in available recordings, providing a sufficient number of seizures for further analysis. Seizure semiology was defined according to semiological classification guidelines (10, 12) for each seizure type, using additional descriptors for the observable movements during a seizure. Seizure semiologies for each patient have been presented in Supplementary material 1.

To optimize the seizure signal analysis and minimize the effect of the background noise of the video event, seizure video clips were cropped from the raw data by a professional epileptologist. Videos were cropped so that they included the seizure onset and the assumed ending of the seizure activity by comparing the seizure manifestations in recorded video events and VEM reports. The postictal phase was left out of the analysis. For each seizure type and patient, the medians of motion, audio and oscillation signal were calculated using the method described in Section 2.3.

### 2.3. Signal generation from video data

The model of the system has been described in the previous proof-of-concept study (24) in detail. Similarly, the system relies on motion and oscillation biomarkers.

To create a motion signal, a background subtraction method by Zivkovic and Van Der Hejden (33) was combined with a stereo correspondence filter (34) based on semi-global matching (implemented in OpenCV). The background subtraction model created a binary mask of the moving parts of the image, and the proportion of the moving pixels in an image defined a one-dimensional motion signal for a video.

For movements with an oscillatory component (as present in tonic-clonic seizures), an optical flow-based method was utilized. By using this method, a time-series motion vector field was created. This vector field was utilized to construct a path history, where only the unbroken paths during a period of 1 s were analyzed for direction reversal (a reversal is each change in direction over 90°). An oscillation frequency of 2.5 Hz was previously found to be a good filter for separating ictal oscillation from paroxysmal events (24).

### 2.4. Clustering analysis

To separate hyperkinetic, tonic, and tonic-clonic seizures, unsupervised data representations were explored. A common technique for data visualization and exploration was used; a cluster diagram where a data sample is represented by a point on a 2D chart that is inspected by a human or a clustering algorithm to find meaningful structures (clusters) to solve a problem. Since the samples are usually multidimensional, they must be transformed by dimensional reduction algorithms into 2D space before drawing the diagram. After the original data are projected into lower dimensions, the diagram axes do not have any particular unit or meaning.

After the complete feature extraction from the patient videos, the time series of motion and oscillation signals were reduced in two dimensions. Each time series was transformed to lower data space by extracting time-series statistical features in order to have low and fixed data dimensions. The seizures had varying duration and variable length time series, but a fixed data dimension for conducting a principal component analysis (PCA) for the data reduction into 2D was used. To analyze ictal motion characteristics in the video data, motion features from the catch22 feature collections were utilized in this study. During the initial experimentation, 22 statistics were calculated by the catch22 library (26) from the training set and fed into PCA. With the final 2D data, cluster plots were created representing the seizures in different colors to visualize their distribution. The discrimination power of 22 statistics was further analyzed on the training set, and the original catch22 feature set was then reduced to five features before the PCA step by visually observing the relatively unchanging cluster diagram after incrementally removing redundant features.

Data clustering can be especially applied for data visualization, but separate training and testing steps were implemented in this experimentation. The dimension reduction methods were first used for the training patient group to develop an initial visualization and to find the most optimal parameters for seizure differentiation. After the training phase, the computed PCA coefficients were applied to the testing patient data for projecting the testing data points by the same dimension reduction transformations into the 2D data space and assessing the performance of the model by visual evaluation of data points and then by classification analysis discussed below. In the final step, agglomerative clustering was used to discover clusters on the image and observe how the unsupervised cluster represents the different seizure types.

### 2.5. Classification analysis

Unlike the unsupervised clustering analysis that is based on dimension reduction and cluster identification on 2D plots, a classification method was also employed in this study to assess the performance of a supervised learning approach. A better insight can be given to the discriminative power of the extracted features by analyzing the same data using these different techniques because the first method reduces the data dimension drastically while the second method works on the time-series directly. Note that the time-series of the pixel statistics are already a heavily reduced data dimension compared to the original video frames. A deep-learning network (multivariate long short-term memory with fully connected layers—MLSTM-FCM) specialized for time-series classification (35) was built on the training set to classify the data points of hyperkinetic, tonic, and tonic-clonic seizures and make predictions for unseen data points of the testing set. The implementation was based on the tsai library (36). The hyperparameters were an RNN layer count of 2, a hidden neuron count of 200, and RNN and FCN dropouts of 0.05. The previous clustering method transformed the time series into 2D data with dimensional reduction techniques (catch22, PCA), and the MLSTM-FCN model worked on the time series directly, processing and classifying a time series into a single seizure category.

**Figure 1:**
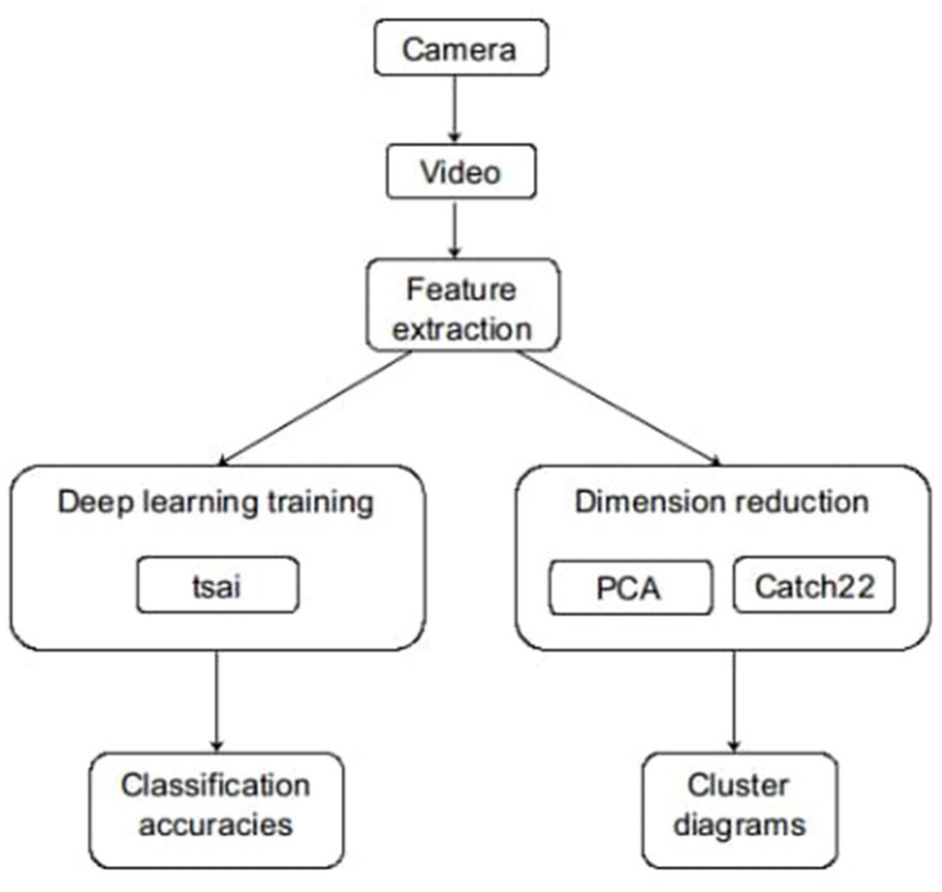
Summary of the method.

After the automatic analysis of the data points of the testing set, we evaluated the performance of the deep-learning network by calculating the accuracy of the classification of hyperkinetic, tonic, and tonic-clonic seizures. Based on the classification, the overall accuracy of the model was determined. The description of the method used in this study is presented in Figure 1.

## 3. Results

### 3.1. Unsupervised clustering analysis

**Figure 2:**
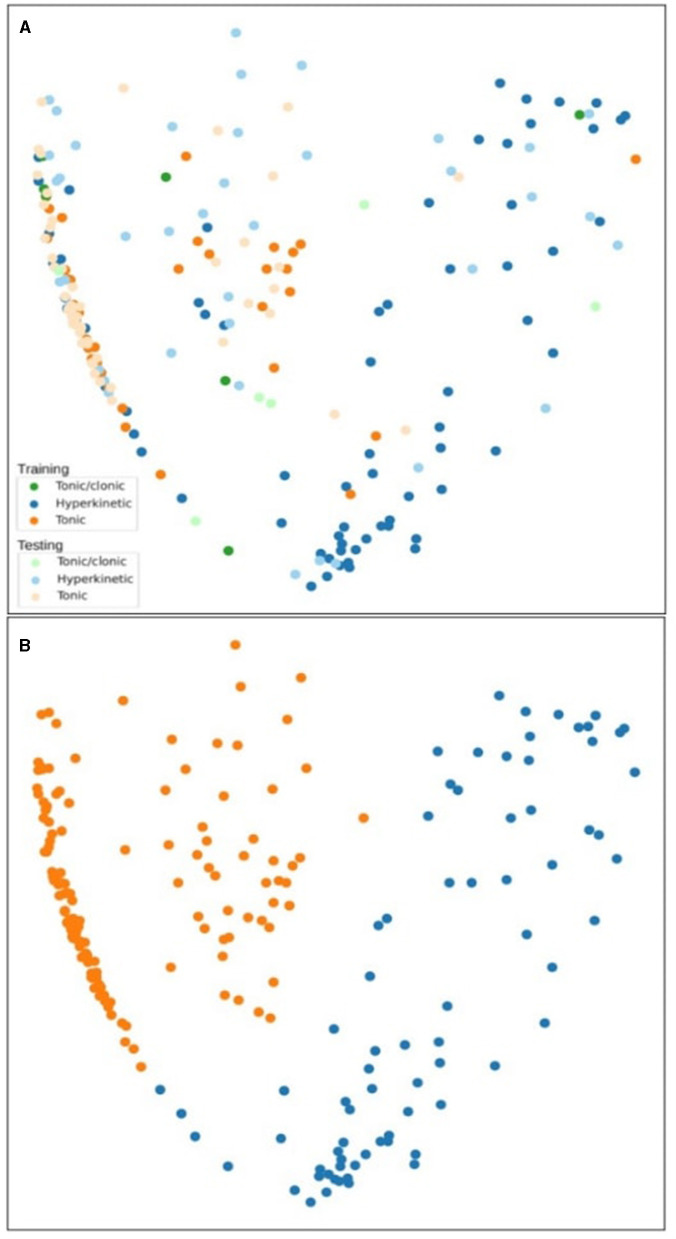
Clustering analysis of tonic-clonic, hyperkinetic, and tonic seizures using static motion features in the training and testing phase **(A)**. The second figure shows the agglomerative clustering results **(B)**.

**Figure 3:**
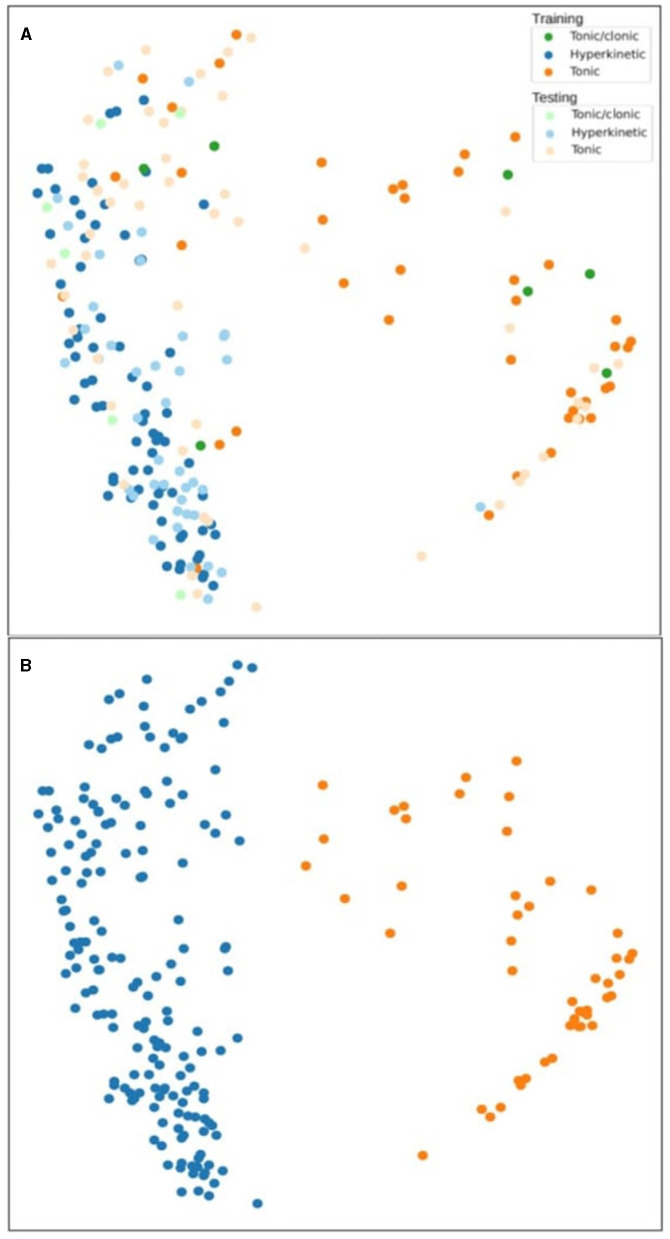
Clustering analysis of tonic-clonic, hyperkinetic, and tonic seizures using temporal motion difference features in the training and testing phase **(A)**. The second figure shows the agglomerative clustering results **(B)**.

Adjunctive changes in the feature generation enabled improved discrimination power to differentiate between tonic-clonic, hyperkinetic, and tonic seizures. Two different motion feature setups, static motion features (Figure 2) and temporal motion features (Figure 3), were used to compare the feasibility of the features. Oscillation tracking was not included in these figures, as it did not improve the seizure cluster formation in clustering analysis (see Supplementary material 2). Although the data points are grouped in different shapes in this case compared to Figure 3, the general considerations do not change, and many tonic-clonic points and a considerable fraction of the clonic points appear in the hyperkinetic cluster.

The static motion features are the original time series extracted from the videos while the temporal features are a delta derivative time series calculated by a lag (a fixed duration, e.g. 1 s), often referred to as time-series lag difference or delta function measuring the change over time. Delta values were calculated with a difference between the current value and a past value (e.g., 1 s before). In all figures, tonic-clonic, hyperkinetic, and tonic seizures were marked as green, blue, and orange, respectively, indicating training phase results, and light green, light blue, and light orange, respectively, indicating testing phase results.

In Figure 2A, a cluster of tonic-clonic and tonic seizures appeared on the left side and hyperkinetic seizure clusters appeared on the right side of the figure. This cluster is visually noticeable in the training phase, while the hyperkinetic cluster spreads to both sides of the figure in the testing phase. Different types of seizures were interspersed in the center of the figure, and hyperkinetic seizures were infused in the left cluster among the tonic-clonic and tonic seizures. The tonic-clonic seizure does not represent a coherent, separate structure among the data points. Figure 2B shows unsupervised clustering results and the agglomerative clustering isolated the tonic and hyperkinetic clusters successfully on the left and right sides.

In Figure 3A, the clusters switched sides: hyperkinetic and tonic clusters were clearly separate, but the majority of tonic-clonic seizures were part of the hyperkinetic seizure cluster region. The hyperkinetic seizure cluster was plotted clearly on the left side of the figure in both the training and testing phases. The tonic seizure cluster was more dispersed in the testing phase than in the training phase. Tonic-clonic seizures did not separate from hyperkinetic seizures in either of the phases when this motion feature was used. In Figure 3B, the agglomerative clustering discovered two clusters, one for hyperkinetic and one for tonic seizures. The tonic seizures are spread above the hyperkinetic seizures on the left side and the clustering was not able to include this upper part in the tonic cluster.

### 3.2. Performance analysis

To analyze the performance of the method, incremental analysis was done in addition to unsupervised clustering analysis. By training a deep-learning network based on the background subtraction signal and comparing the results with original annotations, we calculated the accuracy of the seizure classification method. We ran a leave-one-out cross-validation of the deep-learning method. The cross-validation was then repeated 100 times to calculate an estimation of the unbiased accuracy and its confidence interval since the model performance has some variability between each training run because the deep-learning training is not deterministic with the random weight initialization. Our method achieved an overall mean accuracy of 74.68% and an f1-score of 74.26%. The hyperkinetic, tonic, and tonic-clonic seizures had mean accuracies of 91.03, 87.90, and 45.12%, respectively. The mean f1-scores were 92.83, 89.79, and 37.18%, respectively. The hyperkinetic and the tonic seizures had very similar accuracy and f1-score values, while the f1-score of the tonic-clonic seizure was lower by 8%, compared to the accuracy value. The accuracy and f1-scores of hyperkinetic and tonic seizures were high by approximately 90% while the tonic-clonic seizure had only 45.12 and 37.18%, respectively. Regarding the confidence intervals (*p* = 0.05), the hyperkinetic, tonic, and tonic-clonic seizures had 1.1, 1.5, and 4.2% for accuracy and 1, 1.5, and 4.1% for f1-scores, respectively. The confidence intervals were similar for hyperkinetic and tonic seizures, but more than double for tonic-clonic seizures. As the low accuracy and its high confidence interval of tonic-clonic seizures suggest, this seizure type was not recognized on a satisfactory level because of the lack of enough patient data to distinguish this seizure type from the other two types. This result is on pair with our unsupervised clustering results where the hyperkinetic and tonic seizures can be separated quite well, but the tonic-clonic data points are spread around. The accuracy and confidence interval of each seizure type are presented in Table 2.

**Table 2:** The unbiased accuracy, f1-scores, and confidence intervals after 100 cross-validation runs.

**Unbiased accuracy, f1-scores, and confidence intervals after 100 cross-validation runs**			
	**Hyperkinetic seizures**	**Tonic seizures**	**Tonic-clonic seizures**
Mean accuracy	91.03%	87.90%	45.12%
Confidence intervals for accuracy	±1.1%	±1.5%	±4.2%
F1-score	92.83%	89.79%	37.18%
Confidence intervals for f1-score	±1%	±1.5%	±4.1%

## 4. Discussion

In this study, we present a novel method for differentiating between tonic-clonic, tonic, and hyperkinetic motor seizures based on the automatic analysis of motion and oscillation signals from previously annotated video data. This algorithmic component of Nelli^®^ hybrid (algorithmic-human) seizure monitoring system has been previously studied for automated seizure detection, but in the present study, it was tested as an automated seizure classification tool by applying adjunctive video and unsupervised clustering analysis for the first time. We intended to develop the differentiation algorithmic ability that would aid clinicians in classifying seizures for Nelli^®^ hybrid seizure monitoring, which is currently used in clinical practice (37). In the present study, our model differentiated and classified hyperkinetic and tonic seizures with a promising accuracy of 91 and 88%, respectively. However, tonic-clonic seizures were classified with only 45% accuracy. The f1-scores for hyperkinetic, tonic, and tonic-clonic seizures were 93, 90, and 37%, respectively.

Screening and differential diagnosis between different seizure types are essential components in the detection of seizures and the correct implementation of treatment (4). Seizure classification relies on objective criteria of ictal observations of caregivers or clinicians. Most motor seizures have distinguishable motor manifestations that indicate a specific seizure type; however, oftentimes, there are no eyewitnesses, especially, for nocturnal seizures (4). However, depending on the motor manifestations of seizures, seizure type classification can be difficult, even with the help of seizures recorded on videos because seizure semiology is often prone to inter-observer discrepancy due to qualitative criteria reliance (observer bias) (38). Moreover, it is very time- and resource-consuming to manually annotate and classify seizures of each patient (39, 40). A system capable of measuring seizure features qualitatively as well as quantitatively would allow the detection of changes in seizure severity or seizure propagation. Also, in case of multiple seizure types and high seizure frequency during the monitoring period, automatic tools could be useful to save time and resources in video annotation during seizure monitoring. Automatic classification could also improve seizure alarm systems by enabling alarms for different seizure types, which might be useful, especially in epilepsy monitoring units or institutional settings. Furthermore, EEG-based automatic seizure classification methods have already been examined with promising results as a clinical application of the automatic seizure classification tool (41).

In previous studies, automatic classification of epileptic seizures from psychogenic non-epileptic events was conducted by a multi-stream approach, reaching f1-scores and accuracy of 0.89 and 0.87, respectively, in seizure-wise cross-validation and 0.75 and 0.72, respectively, in leave-one-subject-out analysis (42). Hyperkinetic seizures have been automatically differentiated from non-hyperkinetic seizures and sleep-related paroxysmal events with 80% probability (43) and 80% accuracy (44). In another study, CNNs and recurrent neural networks (RNNs) were combined to automatically classify seizure videos into focal onset seizures and focal to bilateral tonic-clonic seizures achieving 98.9% accuracy (45). However, an automatic system that differentiates motor seizures into three types has not been previously reported in the literature. Our study reached a relatively good accuracy in hyperkinetic and tonic seizure classification, and the results from hyperkinetic seizure classification aligned with the previous research study (43). However, tonic-clonic seizures were not differentiated as accurately as the other two seizure types, which weakens the performance, especially when considering the clinical relevance of tonic-clonic seizure documentation in decreasing the risk of SUDEP (46). However, in previous validation studies of the Nelli^®^ seizure monitoring hybrid (algorithmic-human annotation) system, all tonic-clonic seizures were correctly categorized (23) due to stereotypic and easily recognizable motor manifestations. Since the tonic-clonic seizures cannot be separated in both the clustering and the classification methods in this study, it suggests that this limitation is not caused by the applied methods, but the extracted time-series descriptor does not have discriminative power for this task.

Catch22 was utilized to extract statistical descriptors to reduce the data dimensionality of the training and testing sets drastically. This library turned out to be suitable for this task as a collection of the best statistics for time-series analysis across various science fields. To select the statistical features with real discriminative power for the current study, the redundant steps were removed from the initial set one by one after inspecting the unaffected cluster diagrams. The deep-learning experiment after the cluster analysis confirmed the good overall discriminative power. Since there were few tonic-clonic seizures compared to hyperkinetic and tonic seizures, they were not distinguished as well as the other seizures. This is not a by-product of catch22 or deep learning, but a common phenomenon in machine learning when a class is underrepresented in the learning task.

This study has several limitations. Our patient population was quite small, and especially the number of tonic-clonic seizures included in the study was low. The majority (>90%) of seizures included in the study consisted of hyperkinetic and tonic seizures, which may have affected the performance of our model, and a dataset with more evenly represented seizure types might improve the model development in future research. Also, due to availability, we only included tonic, tonic-clonic, and hyperkinetic seizures, which usually have recognizable motor manifestations and are reliably classified by human annotators. Seizures included in this study represented varying semiologies even within a seizure type, as shown in Supplementary material 1, which may have caused challenges in classification. A larger patient group would enable further training of the model and improve the statistical reliability of the results. However, running cross-validation 100 times as done in our study was found as one solution to this issue. The patient dataset consisted of adult patients, which limits the generalizability of the results to pediatric patients. Furthermore, we only tested seizures that were confirmed to be seizures, and we did not have a category for non-epileptic events involved, which may cause an overestimation of the performance. However, previous studies that utilized our system have shown the accuracy of automatic seizure detection in various seizure types (43). The exclusion of non-epileptic events from hyperkinetic seizures has been also reported with relatively high accuracy (42). In addition, seizures with more subtle motor manifestations can be challenging to detect automatically, as previously reported (23), even though studies have shown accurate detection for those seizures (16). However, seizures with minor motor manifestations may be difficult to detect, even for human annotators, which provides a topic for future development. Furthermore, simultaneous analysis of more seizure types might be challenging due to the similar motion and oscillation signal profile such as myoclonic and tonic seizures, as well as tonic-clonic and clonic seizures. There are other general limitations related to video monitoring: a patient should stay in sight of the camera, a caregiver should avoid being in the frame of the camera to not affect the motion signal, and a blanket can impede the movement of the patient. It is important to maintain the same monitoring settings throughout the monitoring period to avoid the effect of patient and environment-related factors on movement detection (47).

## 5. Conclusion

The quantitative analysis with selected motion features distinguished semiological differences between epileptic motor seizures and enabled differentiation of hyperkinetic and tonic seizure types from video data in patients with DRE. Our results suggest that the motion signal profiles seem to allow motor seizure differentiation and classification. The system achieved a promising accuracy and f1-score of 74% in the testing phase. Tonic and hyperkinetic seizures were classified with 91 and 88% accuracy, respectively, but accuracy for tonic-clonic seizures was only 45%. The f1-scores of hyperkinetic, tonic, and tonic-clonic were 93, 90, and 37%, respectively. Future studies are needed with a larger and more robust dataset, including additional motor seizure types and false positive events. These developments hold the potential to streamline the clinical workflow of video-based seizure monitoring systems by providing a supporting tool for seizure classification. In summary, despite the lack of accuracy in the classification of tonic-clonic seizures, the results from the present study can be considered a step forward toward an automatic seizure classification tool for clinicians.

## Data availability statement

The raw data supporting the conclusions of this article will be made available by the authors, without undue reservation.

## Ethics statement

The studies involving humans were approved by Ethics Committee of Tampere University Hospital. The studies were conducted in accordance with the local legislation and institutional requirements. Written informed consent for participation in this study was provided by the participants' legal guardians/next of kin. Written informed consent was obtained from the individual(s), and minor(s)' legal guardian/next of kin, for the publication of any potentially identifiable images or data included in this article.

## Author contributions

PO: Writing—original draft, Writing—review & editing. CK: Writing—original draft, Writing—review & editing, Formal analysis, Software. EM: Formal analysis, Software, Writing—review & editing. PR: Formal analysis, Writing—review & editing. KA: Project administration, Writing—review & editing. AK: Data curation, Formal analysis, Investigation, Methodology, Writing—review & editing. JP: Data curation, Investigation, Supervision, Writing—original draft, Writing—review & editing.
