# Supplementary material for: Automatic classification of hyperkinetic, tonic, and tonic-clonic seizures using unsupervised clustering of video signals
Source: Front Neurol. 2023 Nov 2;14:1270482. doi: 10.3389/fneur.2023.1270482 (PMC10652877; doi:10.3389/fneur.2023.1270482)
Supplement: Supplementary file 1 [file Data_Sheet_1.docx]

Supplementary material 1. Seizure semiologies for each patient.

| Training patients | | |
| --- | --- | --- |
| Patient number | Seizure type | Seizure semiology |
| 1 | Tonic | Eyes open – tonic BL |
| 2 | Tonic-clonic | Eyes open – tonic BL – clonic BL |
|  | Tonic | Eyes open – tonic BL |
| 3 | Tonic-clonic | Eyes open – tonic BL – head version L – clonic BL |
|  |  | Eyes open - tonic BL - clonic L - dystonic posturing BL |
| 4 | Tonic | Eyes open – tonic BL |
| 5 | Hyperkinetic | Eyes open – hyperkinetic BL - vocalization |
| 6 | Hyperkinetic | Hyperkinetic L – heavy breathing |
| 7 | Tonic-clonic | Eyes open- vocalization – convulsive movement |
| 8 | Hyperkinetic | Eyes open – hyperkinetic BL - vocalization |
| 9 | Hyperkinetic | Eyes open – hyperkinetic BL - vocalization |
| 10 | Hyperkinetic | Eyes open – hyperkinetic BL - vocalization |
|  | Tonic-clonic | Motor BL - tonic BL – head version L – clonic BL |
| Testing patients | | |
| Patient number | Seizure type | Seizure semiology |
| 1 | Tonic-clonic | Head version L – Tonic BL – clonic BL |
| 2 | Tonic-clonic | Tonic BL – head version R – clonic BL |
| 3 | Tonic-clonic | Tonic BL – vocalization – head version L – clonic BL |
| 4 | Tonic | Motor BL – tonic BL |
| 5 | Tonic | Eyes open – tonic BL |
| 6 | Tonic | Tonic BL – eyes open – head version L |
| 7 | Tonic | Tonic BL |
| 8 | Tonic | Tonic BL – vocalization |
| 9 | Tonic | Tonic BL – eyes open |
| 10 | Tonic | Tonic BL – eyes open - vocalization – head version R |
| 11 | Hyperkinetic | Eyes open – hyperkinetic L |
| 12 | Hyperkinetic | Motor BL – hyperkinetic BL |
| 13 | Hyperkinetic | Arousal – facial contraction BL – Hyperkinetic BL |
| 14 | Hyperkinetic | Hyperkinetic BL - vocalization |
| 15 | Hyperkinetic | Hyperkinetic BL – eyes open - vocalization |
| 16 | Hyperkinetic | Hyperkinetic BL – vocalization |
| 17 | Hyperkinetic | Eyes open – hyperkinetic BL - vocalization |
