# Supplementary material for: Automatic classification of hyperkinetic, tonic, and tonic-clonic seizures using unsupervised clustering of video signals
Source: Front Neurol. 2023 Nov 2;14:1270482. doi: 10.3389/fneur.2023.1270482 (PMC10652877; doi:10.3389/fneur.2023.1270482)
Supplement: Supplementary file 2 [file Data_Sheet_2.pdf]

Supplementary material 2

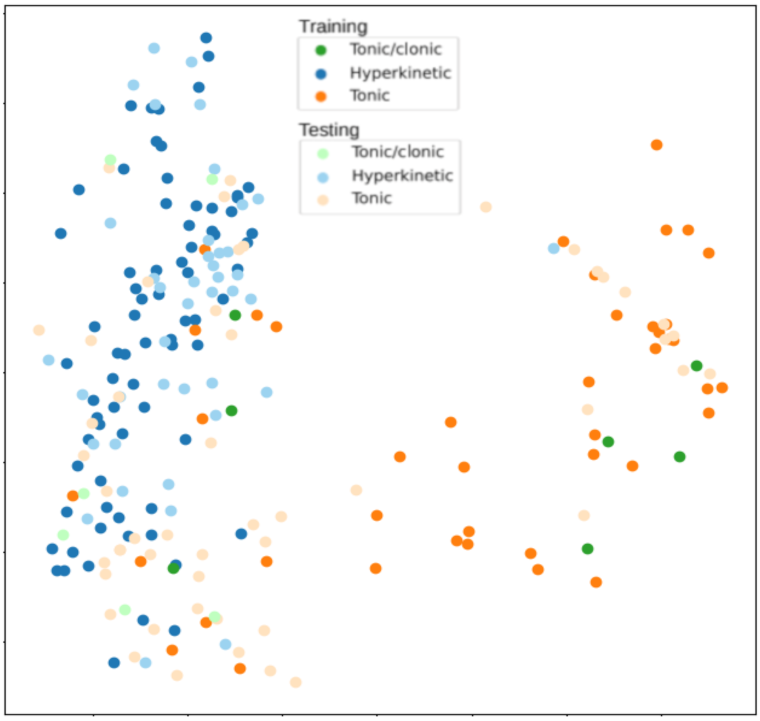

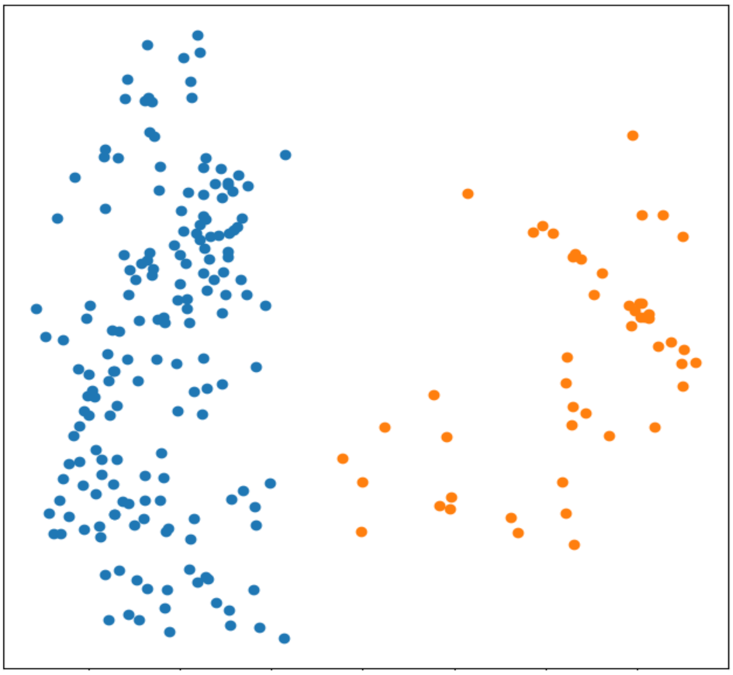

Supplementary material 2. Clustering analysis of tonic-clonic, hyperkinetic, and tonic seizures using a combination of temporal motion features and oscillation tracking in the training and testing phase. The second figure shows the agglomerative clustering results.
